# Investigating resilience in patients with IBD: preliminary insights for understanding disease-specific resilience skills

**DOI:** 10.3389/fpsyg.2024.1486401

**Published:** 2024-11-13

**Authors:** Michelle Mendiolaza, Tonia Ogundipe, Juan Arroyave-Villada, Olusola Adeonigbagbe, Ksenia Gorbenko, Laurie Keefer

**Affiliations:** ^1^Institute for Translational Sciences, Icahn School of Medicine at Mount Sinai, New York, NY, United States; ^2^Medical Education, Icahn School of Medicine at Mount Sinai, New York, NY, United States; ^3^Population Health Science and Policy, Icahn School of Medicine at Mount Sinai, New York, NY, United States; ^4^Institute for Healthcare Delivery Science, Mount Sinai Health System, New York, NY, United States; ^5^Division of Gastroenterology, Icahn School of Medicine at Mount Sinai, New York, NY, United States

**Keywords:** inflammatory bowel diseases, psychological resilience, patient-centered research, quality of life, positive psychology

## Abstract

**Introduction:**

Inflammatory bowel diseases (IBDs) significantly impact patients’ quality of life. While research highlights the potential role of *psychological resilience* to enhance overall health and well-being in patients with chronic conditions, its specific role in the context of IBD remains underexplored. This study aimed to identify key components of resilience, while serving as a precursor to the development of a disease-specific Resilience Scale for IBD (RISE-IBD).

**Methods:**

In semi-structured focus groups and individual interviews, fifteen patients with IBD discussed their perspectives on the construct of resilience, particularly in terms of the diverse strategies they employed to overcome IBD-related challenges. Patients also deliberated on the relevance of themes identified in two widely used and validated resilience measures. Four analysts coded the transcripts using MAXQDA. Selected items for the drafted measure were refined based on constructive feedback from an additional focus group with study participants and six multidisciplinary IBD professionals, thus establishing face and content validity.

**Results:**

The qualitative analysis revealed critical themes of resilienc*e* for IBD including: (1) seeking physical and emotional support from others, (2) developing personal coping mechanisms to manage stress, and (3) employing strategic disease-management techniques. These themes led to the identification of 17 items, which were categorized into three domains: interpersonal fortitude, individual character strengths, and logistical strategies.

**Discussion:**

This study highlights the critical role of resilience, a core concept in positive psychology, in the effective self-management of IBD. The findings underscore the importance of building upon resilience strategies to help patients bolster the psychological potencies needed to manage their condition more effectively. Future research will focus on the psychometric validation of items identified for the scale. By incorporating resilience-building strategies into IBD care, we can support patients in developing a more positive outlook and improved life satisfaction.

## 1 Introduction

Inflammatory bowel diseases (IBDs), which encompass Crohn’s disease (CD) and ulcerative colitis (UC), are chronic, inflammatory illnesses that result in unpredictable bowel movements, painful gastrointestinal tract activity, and nutritional concerns (Casellas et al., 2002). With an unknown etiology likely influenced by both genetics and the environment, IBDs affect an estimated 3 million individuals in Europe and 3.1 million in the United States (U.S.), with incidence rates continuing to rise (Dahlhamer et al., 2016).

IBD symptoms that lead to the disruption of daily functioning may not only contribute to an individual’s perceived disability, but could also increase the likelihood of mood disorders, consequently affecting patients’ overall quality of life (Casellas et al., 2002; Sun et al., 2019). Furthermore, the inability to manage such unpredictable disease demands have been linked to mental health disorders and subsequently poor treatment compliance (Cohen et al., 2022). Previous studies showed that integrating psychotherapy targeting anxiety and depression, resulted in decreased healthcare utilization among patients with IBD (Von Wietersheim and Kessler, 2006; Deter et al., 2007). These notions underscore the clinical significance of monitoring psychosocial well-being in conjunction with addressing physical discomfort, emphasizing the relevance of psychological dimensions in effective management of IBD (Robertson et al., 2019).

Resilience, the ability to adapt to adversity physically and emotionally, is a malleable psychological characteristic that is positively responsive to behavioral interventions (Helmreich et al., 2017). Notably, resilience and grit, while related, are not synonymous. Grit refers to a sustained passion and perseverance for long-term goals, whereas resilience encompasses the personal qualities that enable an individual to thrive in the face of hardship (Meyer et al., 2020). A recent study showed that resilience could mediate the relationship between depression, anxiety, and disease activity in patients with IBD (Keefer, 2018; Sehgal et al., 2021). Accordingly, resilience could be leveraged to mitigate psychological disorders such as anxiety in the IBD population, the prevalence of which has been estimated to be as high as 40% (Bannaga and Selinger, 2015).

Presently, there are limited disease-specific resilience measures available such as those developed by Sinclaur and Wallston who assessed individuals with rheumatoid arthritis (Sinclair and Wallston, 2004), Friborg who studied Norwegian mental health outpatients (Friborg et al., 2003), and Thongkhum whose research centered on Thai elderly with chronic diseases and depression (Thongkhum et al., 2022). Furthermore, despite the widespread use of the Connor-Davidson Resilience Scale (CD-RISC) (Connor and Davidson, 2003), a valid self-report tool for assessing resilience in adult populations, it is important to consider that individuals with IBD face unique challenges specific to their condition. Generic resilience tools often contain items that do not reflect the specific experiences of IBD patients, such as managing flare-ups, coping with social stigma, and addressing unique mental health needs. This can lead to inaccurate assessments of disease-specific resilience. Therefore, there is a need for a patient-centered outcome tool that recognizes the dynamic and evolving needs of patients with IBD. Accordingly, healthcare providers could use such a tool to quantify *IBD-related resilience* and measure its ability to change over time or when supported by behavioral interventions.

To date, however, a standardized definition of resilience for patients with IBD has not been established. While psychological resilience may not represent a singular construct, it is likely to encompass varied facets of biopsychosocial function, influenced by internal and external factors (Richardson, 2002; Luo et al., 2019). Indeed, understanding resilience requires acknowledging its multi-dimensional nature, which encompasses a spectrum of psychological, emotional, and social processes, highlighting the need for a robust comprehension of the construct to effectively measure resilience within this population’s context. Patient-led qualitative exploration can assist healthcare professionals in comprehending various aspects of lived patient experiences that are currently overlooked, particularly concerning psychosocial and resilience-based challenges (Gill et al., 2016; Rines et al., 2022).

A unique method for collecting patient-reported outcomes (PROs) involves obtaining information directly from patients, emphasizing the patient’s voice through the development of psychological measures including those for resilience (Bingham et al., 2016; Ruan et al., 2017; Thongkhum et al., 2022). Particularly, the Patient-Reported Outcomes Measurement Information System (PROMIS) emphasizes the use of patient input to develop measures that are relevant and meaningful to patients’ lives. In fact, PROMIS item banks were refined through extensive patient input via focus groups and cognitive interviews, ensuring that the measures reflected patients’ perspectives and were easily understandable (Bingham et al., 2016). Similarly, the Illness Perception Questionnaire-Revised (IPQ-R) (Weinman et al., 1996) utilized qualitative interviews with patients to understand their perceptions of illness, which informed the development of questionnaire items. By seeking patients’ input early in the development process, investigators are able to reduce the length of surveys while maintaining their relevance to the construct of interest (Bingham et al., 2016).

Given the limited research on the definition of resilience for individuals with IBD, the primary objective of this study was to gain insight into the specific characteristics that individuals with IBD recognized as indicative of resilience, achieved through direct conversations with patients. The study intended to utilize definitions of both psychological and IBD-specific resilience to develop a future outcome assessment designed to measure resilience within the context of IBD.

## 2 Materials and methods

A patient-focused qualitative study, consisting of three sequential focus group discussions (FGDs) and one member check, aimed to characterize the concept of resilience in IBD, including associated attitudes, beliefs, and behaviors. The insights gathered from these discussions aimed to inform the development of a new quantitative Patient-Reported Outcome Measure (PROM), called the “Resilience Scale for IBD (RISE-IBD).”

### 2.1 Ethical consideration

This study involving humans was approved by the Institutional Review Board (IRB) at the Icahn School of Medicine at Mount Sinai on October 3, 2022 (Protocol Number: STUDY-22-01251). The study was conducted in accordance with the local legislation and institutional requirements. The participants provided their written informed consent to participate in this study.

### 2.2 Recruitment and study sample

Patients were purposively recruited from a U.S. outpatient IBD clinic following their routine visits and were contacted by phone from November 2022 to June 2023. The inclusion criteria included individuals who were 18 years of age or older, had a confirmed diagnosis of UC or CD for at least 3 months, were able to provide informed consent in English, and could complete the Brief Resilience Scale (BRS) (Smith et al., 2008) with a score of at least 3.00, indicating “moderate level” resilience. This criterion would ensure that individuals could openly articulate the barriers linked to IBD and the strategies employed to overcome them.

Out of the 24 patients who expressed interest in participating, 4 did not meet the BRS criteria and 5 were lost-to follow-up before the start of the first discussion. Therefore, a total of 15 eligible patients who met the BRS criteria participated in the study and self-reported their demographic characteristics (see Table 1). Each of the 15 patients took part in three discussions (i.e., FGDs) either in focus groups or through one-on-one interviews, depending on their availability. This flexible approach was used to accommodate scheduling constraints when participants were unable to meet collectively. Additionally, as part of a member check with stakeholders, six IBD healthcare professionals (including four physicians, one pharmacist, and one dietitian) were invited to provide feedback on the study’s findings.

**Table 1:** Patients’ demographic characteristics.

	Overall (*N* = 15)
Brief Resilience Scale (BRS) score	
Mean (SD)	3.10 (0.50)
Range	2.50–3.83
Age, years	
Mean (SD)	45.5 (16.2)
Range	23.0–78.0
Gender	
Female	10 (66.7%)
Male	5 (33.3%)
Ethnicity	
NOT Hispanic or Latino	13 (86.7%)
Hispanic or Latino	1 (6.7%)
Unknown	1 (6.7%)
Race	
White	11 (73.3%)
Asian	2 (13.3%)
Unknown	2 (13.3%)
Education	
Bachelor’s Degree	7 (46.7%)
Graduate or terminal degree	8 (53.3%)
Marital status	
Divorced	2 (13.3%)
Single	3 (20.0%)
Married/Domestic partnership	10 (66.7%)

The average BRS score of the 15 participants was 3.1 (SD 0.50). The mean age was 45 years (SD 16.2) with age range 23–78 years. Many of the participants were Female (*n* = 10) and non-Hispanic or Latino (*n* = 13). Regarding clinical characteristics (see Table 2), more than half of the participants had Crohn’s disease (*n* = 9). Moreover, 7 of the participants had been living with IBD for 1–5 years, while 8 had lived with IBD for more than 10 years. Most patients (*n* = 12) reported that they were in clinical remission during the study. Considering hospitalizations, 8 participants had experienced at least one IBD-related surgery during their lifetime, and 8 participants were using biologic medications to manage their IBD during the study (see Table 3).

**Table 2:** Patients’ clinical characteristics and reported comorbidities.

	Overall (*N* = 15)
Diagnosis	
Crohn’s disease	9 (60.0%)
Ulcerative colitis	6 (40.0%)
Years lived with IBD	
>10 years	8 (53.3%)
1–5 years	7 (46.7%)
Disease state	
Remission	12 (80.0%)
Active	3 (20.0%)
Annual IBD clinic visits	
1–2	10 (66.7%)
3–6	4 (26.7%)
7 or more	1 (6.7%)
IBD-related surgery	
No	7 (46.7%)
Yes	8 (53.3%)
Bowel resection	
No	11 (73.3%)
Yes	4 (26.7%)
Fistulae repair	
No	12 (80.0%)
Yes	3 (20.0%)
Ileostomy	
No	13 (86.7%)
Yes	2 (13.3%)
Colostomy	
No	14 (93.3%)
Yes	1 (6.7%)
Fatigue	
No	7 (46.7%)
Yes	8 (53.3%)
Arthritis	
No	9 (40.0%)
Yes	6 (60.0%)
Depression	
No	12 (80.0%)
Yes	3 (20.0%)
Anxiety	
No	6 (40.0%)
Yes	9 (60.0%)
Psoriasis	
No	11 (73.3%)
Yes	4 (26.7%)

**Table 3:** Medications used by patients during the study.

	Overall (*N* = 15)
Anti-depressant	
No	13 (86.7%)
Yes	2 (13.3%)
Anti-anxiety	
No	13 (86.7%)
Yes	2 (13.3%)
5-Aminosalicylic acid	
No	11 (73.3%)
Yes	4 (26.7%)
Anti-tumor necrosis factor	
No	12 (80.0%)
Yes	3 (20.0%)
Entyvio	
No	12 (80.0%)
Yes	3 (20.0%)
Stelara	
No	12 (80.0%)
Yes	3 (20.0%)
Skyrizi	
No	13 (86.7%)
Yes	2 (13.3%)

### 2.3 Data collection

All patients consented to their interviews being audio-recorded and taking place on the institutional HIPAA-compliant ZOOM platform. Identifying information was removed from the transcripts to ensure patient anonymity. Each FGD and member check was led by a clinical research Ph.D. candidate [M.M.] experienced in focus group facilitation and qualitative research methods. FGDs used a semi-structured topic guide and lasted approximately 60 min each. In the first FGD, each patient was asked nine open-ended questions to explore the impact of IBD on the patients’ lives by understanding the emotional, physical, and mental challenges posed by living with IBD, as well as the strategies patients used to overcome related obstacles (i.e., resilience). A list of the open-ended interview questions is presented below:

Could you share any positive experiences or challenges you have encountered while living with IBD?
How have you managed to recover from the difficulties presented by IBD?
What personal strengths or qualities do you have that help you overcome IBD-related challenges?
Think of someone, whether they have IBD or not, who you consider resilient. In what ways do they demonstrate their resilience?
What influences or motivates your decisions to overcome such challenges that arise with having IBD?
Have other people, specific resources, or strategies been beneficial in helping you handle IBD-related challenges? Please feel free to describe any problem-solving or coping strategies that have been effective.
What does “resilience” mean to *you*? When do you feel most resilient?
Please share an example of a time when you demonstrated resilience. What thoughts, feelings, or emotions did you use to overcome such hardships?
(a) Do you believe resilience is a trait that someone can learn or develop? (b) If so, what advice would you give to a friend with IBD to help them build their resilience?

**Figure 1:**
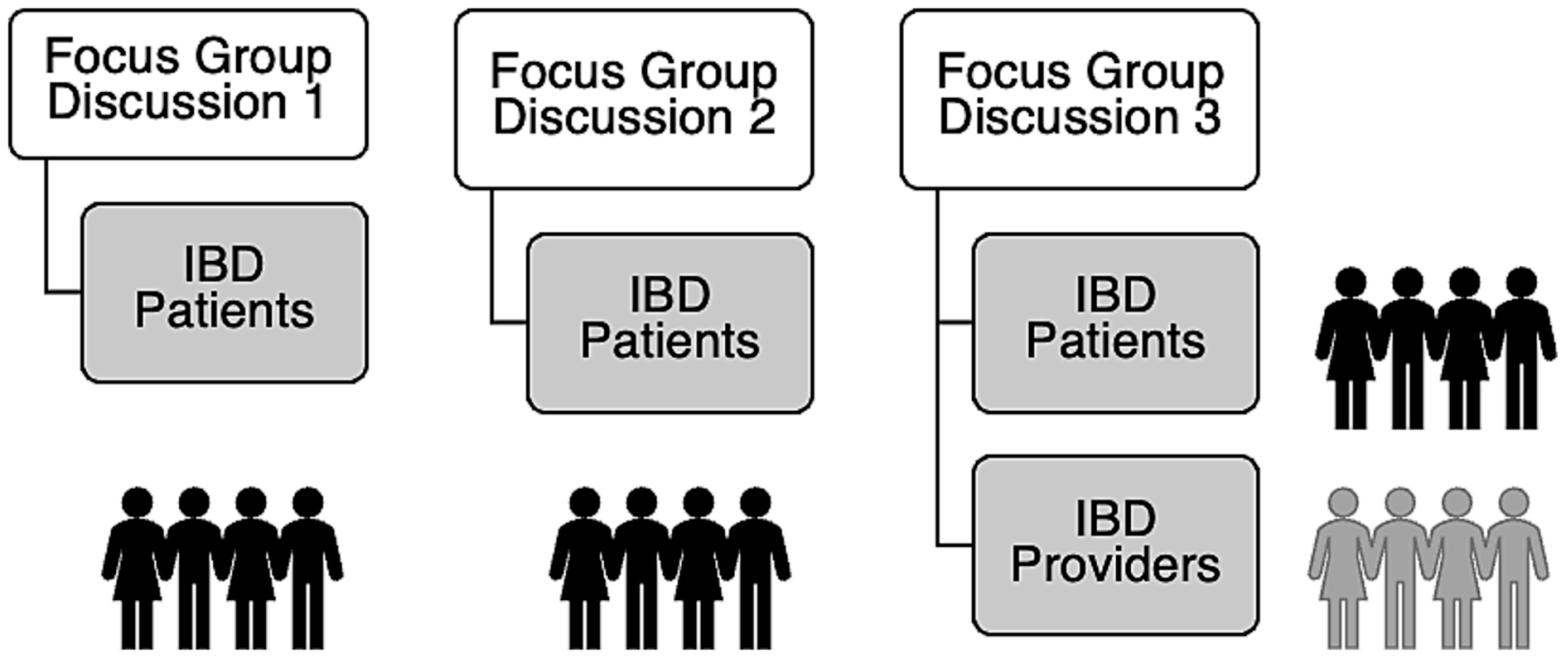
Diagram of the participating groups for each focus group discussion (FGD) with FGD 3 held separately for patients and providers.

In the second FGD, each patient was presented with two validated resilience measures, the CD-RISC (Connor and Davidson, 2003) and the Resilience Scale (Wagnild and Young, 1993), and asked to rate the applicability of themes portrayed in these scales. This approach allowed the researchers to understand how accurately these measures reflected the unique challenges experienced by patients with IBD. Conducting FGDs 1 and 2 sequentially provided patients with ample time to address questions pertinent to each discussion without feeling rushed, therefore allowing the research team to carefully capture participants’ opinions comprehensively. After completion of FGDs 1 and 2, the research team performed thematic analysis to formulate a list of preliminary items that could be included in the RISE-IBD. For the third FGD, patients and IBD professionals were separately invited to provide feedback on the drafted measure in two distinct sessions. A representation of the focus group discussions and the parties involved are presented in Figure 1.

Following each FGD, research personnel [T.O., J.A.V., and O.A] debriefed with the moderator to discuss field notes taken and explore themes that initially emerged. The debriefing sessions allowed the team to align their interpretations of the discussions and ensure a thorough understanding before proceeding to the formal analysis of the transcripts.

### 2.4 Data analysis

An inductive approach, widely used in social science research, was used to analyze de-identified interview transcripts and establish connections between the research objectives and raw findings without relying on *a priori* expectations (Thomas, 2006). The analysis also used influences from the Grounded Theory (Thomas, 2006; Chun Tie et al., 2019) in which the first step was *open coding*, a data-driven process that involved meticulous inspection of the transcripts to formulate conceptional codes. The primary researcher [M.M] listened to the 22 recordings from FGDs 1 and 2 repeatedly and transcribed them verbatim. These recordings included 2 group interviews and 10 individual interviews for the first session (FGD 1), and 3 group interviews and 7 individual interviews for the second session (FGD 2).

Four coders [M.M, T.O. J.A.V., and O.A.] conducted independent and parallel coding of the transcripts using the qualitative analysis software MAXQDA 2022 (VERBI Software, 2021) by analyzing text line-by-line. Following evaluation of each transcript, the research team met to discuss codes and iterative reflection was used to re-code segments upon consensus. The team monitored data saturation by continually assessing when no new codes or insights emerged from the interview transcripts. Once it was observed that no new codes were contributing novel information, we concluded the coding process. This approach ensured that we captured a thorough understanding of participants’ experiences without redundancy.

*Axial coding* was used to draw connections between codes and form them into themes. Conclusively, *selective coding* was used to link the themes and form then into domains that summarized the fundamental construct of resilience in IBD. In this study, a theme relates to key aspects of participants’ experiences and perspectives and offers insights derived from patterns across related codes. In contrast, a domain refers to a broad, overarching category that organizes related themes and provides a structural framework for understanding the major areas of inquiry within the data.

A comprehensive codebook was developed by three researchers [M.M., L.K., and K.O.] including a PhD candidate, a distinguished professor in psychiatry and medicine with expertise in the development of IBD assessments, and a qualitative medical sociologist. For the purpose of scale development, the selected themes were transformed into scale items. The resulting codebook comprised 15 potential scale items that aimed to provide a systematic framework for categorizing the data obtained during the analysis, ensuring a nuanced understanding of the resilience construct for patients with IBD.

### 2.5 Face and content validity

To establish face and content validity of the potential scale items selected for a future questionnaire, a comprehensive feedback approach involving patient perspectives and insights from six healthcare providers was implemented. The member check with healthcare providers were conducted through one-to-one interviews, using the institution’s HIPAA-compliant Zoom software, and were audio-recorded.

In the third FGD, participants were asked to evaluate the degree to which each item reflected their pre-conceived concepts of resilience as it related to IBD self-management. Additionally, they were asked to assess the clarity and readability of instructions provided for the drafted measure. Participants also provided feedback on Likert scaling, with the 5-level Likert rating method emerging as the preferred choice. Six healthcare professionals with expertise in the field of IBD were engaged to further ascertain the accuracy of the proposed components comprising the resilience construct within IBD. Leveraging their professional insights and experiences, detailed feedback was obtained on whether items were pertinent for inclusion. The continuous input from both patients and healthcare providers led to substantial adjustments including modification of the wording of items, addition of a temporal component to the instructions, and the inclusion of two more scale items.

**Figure 2:**
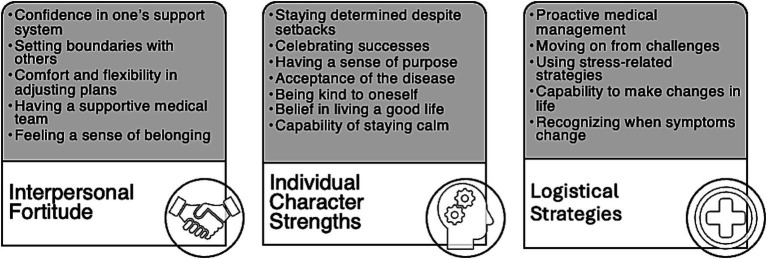
Three identified domains that characterize the attributes of resilience in patients with inflammatory bowel disease (IBD).

This collaboration resulted in the development of a drafted RISE-IBD with 17 items categorized into 3 domains: (1) interpersonal fortitude, (2) individual character strengths, and (3) logistical strategies. The 17 items included in the drafted RISE-IBD were derived from qualitative themes identified during the data analysis. To provide a structured approach for scale development, these items are grouped into 3 overarching domains that represent broader constructs within resilience. Figure 2 presents the 17 items derived from the qualitative themes and their organization under 3 key domains. Instructions on the scale ask participants to reflect on the strategies they use to manage their IBDs when responding to the measure. Responses are given utilizing a 5-point Likert scale ranging from 0 (“not true”) to 4 (“always true”). Scores on the preliminary measure range from 0 to 68, with higher scores indicating greater resilience.

### 2.6 Trustworthiness

Various methods were used to establish the rigor of the findings. According to Guba and Lincoln, the four prominent types of trustworthiness in qualitative research include: credibility, transferability, dependability, and confirmability (Lincoln and Guba, 1986; Thomas, 2006). Credibility, or the internal validity of the study, was achieved by asking participants to verify interpretations that were collected in earlier interviews. The prolonged engagement of the FGDs (60 min each) also allowed the moderator to build trust with participants, resulting in authentic conversations. Transferability, the extent to which the findings can be generalized, was established by meticulously describing the research setting, methods, sample characteristics, and by providing verbatim quotations to enhance the transparency of the data. Dependability, the consistency of the findings, was established through independent and parallel coding. This approach helped identify a robust set of codes and allowed for comparisons to eliminate any team discrepancies in interpretations. Confirmability, the extent to which the findings are shaped by the participants and not researcher bias, was established by allowing participants and the member check to thoroughly evaluate the drafted measure and to confirm or critique the researchers’ findings.

## 3 Results

Three domains emerged from the qualitative study that encompass the multidimensional nature of resilience in IBD patients: (1) interpersonal fortitude, (2) individual character strengths, and (3) logistical strategies to enhance resilience presented in Figure 2. Sample quotes corresponding to each resilience domain are listed in Table 4.

**Table 4:** Resilience domains, themes, and corresponding quotes.

Domain	Theme	Corresponding quote
Interpersonal Fortitude	Confidence in one’s support system	1. “My mom is always coming to the infusions with me, or my husband comes with me and takes off from work. If I did not have such an amazing support system, I do not know how I would overcome everything that comes with IBD.” (FGD1_M)
	Setting boundaries with others	2. “It was making me come to the realization that this was not a healthy relationship for me. That ultimately led to me getting out of the relationship, and two weeks later, my flare started easing up.” (FGD1_S) 3. “Other people’s emotions are huge triggers. Their company is nice but it’s counterproductive because energy is very real and others’ energy rubbing off on you is always going to affect you.” (FGD1_J)
	Comfort and flexibility in adjusting plans	4. “It’s not a losing philosophy if you modify your goals or if you are in a situation and you have to cut back a little bit… people should be educated about that and not picture it as some kind of weakness, but a strength.” (FGD1_C)
	Having a supportive medical team	5. “The doctors that I’ve been working with [in the clinic] have been a really positive experience. I definitely struggled in the beginning with the diagnosis and kind of accepting it, but they really made me feel comfortable with this journey.” (FGD1_M)
	Feeling a sense of belonging	6. “I would join support groups on Facebook or through the Crohn’s and Colitis Foundation. I did the Crohn’s and Colitis Walk recently and knowing that all those people have had similar experiences… it was very empowering.” (FGD1_M) 7. “Trying to find social groups and make online connections made a huge difference. When I connected with people with IBD on Instagram, I found a huge worldwide community and that made a big difference for me.” (FGD2_S)
Individual character strengths	Staying determined despite setbacks	8. “When I remember I have objectives or commitments, I push past the pain. I like to honor the things I promise to do… I feel this makes me a stronger person.” (FGD1_B)
	Celebrating successes	9. “Basically, I say I’m going to accomplish something and then afterwards I can appreciate it. I do not know how I did it. But in the moment, you just keep going. When I reflect on that, I’m very proud.” (FGD1_F) 10. “Just the gift of being able to get up every morning is something I’ve been very appreciative of. If you can find gratitude, you’ll find reasons to want to fight [your IBD] that has its foot on your neck sometimes.” (FGD1_A)
	Having a sense of purpose	11. “I give my life meaning and I think that my IBD has given me a pretty clear-cut goal recently, which has just been to be healthy. That’s sort of been my holy grail purpose.” (FGD2_F)
	Acceptance of the disease	12. “Radical acceptance helped me accept my diagnosis. [In the book] he is like, so you have Crohn’s disease, now what are you going to do - sit there and cry about it? That harshness and reality was such a lifesaver for me.” (FGD1_S)
	Being kind to oneself	13. “I think the concept of taking things in stride is really about being patient and understanding [your disease]. Something to take from it is self-compassion and slowing down.” (FGD2_S)
	Belief in living a good life	14. I feel like I can either succumb [to the disease] or I can try to fight it. And some days it’s hard and I feel bad for myself, but the desire to want to live as normal a life as possible, it’s not even a mindset. (FGD1_A)
	Capability of staying calm	15. “Knowing how to react to situations and staying calm is important because if you do not stay calm it could really flare up your Crohn’s. Lots of times I could be having pain and when I see the doctor they say, how are you so calm? I say, I’ve learned to stay calm.” (FGD1_B)
Logistical strategies	Proactive medical management	16. “I think it’s just so important to be in frequent contact with your doctor. Do not self-medicate. Get advice from the medical experts… I cannot emphasize enough the change that Stelara has made for me. I do not know how I would be if I was still relying on previous medications.” (FGD1_B)
	Moving on from challenges	17. “Finally getting into remission and then having a bad flare, and then recovering again… that [process] really helped get me through the next flare because then I knew that recovery was possible.” (FGD1_L)
	Using stress-related strategies	18. “I’ve meditated for many years, and I think that is the single most important thing for me, developing some kind of [mental] balance. Physically, I do not think there’s anything I’ve been able to take to manage my condition necessarily, but mentally, I’ve been able to not react and get frustrated.” (FGD1_P)
	Capability to make changes in life	19. “I have to eat much less red meat than I used to eat. I can have virtually no fried foods, except for sometimes French fries. I know that if I drink too much alcohol, that will trigger some response with my gut. Eating out is difficult… so I have to be careful about that.” (FGD1_B)
	Recognizing when symptoms change	20. “Sometimes you have to stay near a bathroom after you have eaten. I’ve noticed that after I eat, I really cannot go anywhere… I have to adapt my schedule to when I think [an accident] will happen. Because if you do not time it properly, or if there’s no restroom around, you could be in a very unpleasant situation.” (FGD1_B)

### 3.1 Interpersonal fortitude in IBD

#### 3.1.1 Confidence in one’s support system

Participants expressed a high level of confidence in their support systems consisting of caregivers, family, and friends to provide both emotional and physical assistance. Particularly, participants expressed assurance in being able to rely on direct family members to provide emotional encouragement and guidance with managing the demands of IBD-related events such as unexpected flare-ups or medical appointments.

#### 3.1.2 Setting boundaries with others

Participants emphasized the importance of communicating their needs and limitations to others, whether it was in a professional setting or social outing, resulting in improved relationships and reduced stress. Remarkably, a few patients expressed the act of recognizing when to remove unsettling relationships from one’s life, resulting in a change to patients’ perceived disease status further contributing to their overall well-being.

Moreover, participants believed that the attitudes of others regarding their IBD played a significant role in influencing their emotional well-being. They expressed a heightened sensitivity to the sentiments of those in their immediate environment, emphasizing how such emotional dynamics could affect them personally.

#### 3.1.3 Comfort and flexibility in adjusting plans

Many participants expressed the importance of being comfortable changing plans based on the status of their condition, such as during periods of flare-ups. This portrayed an adaptive approach to managing their interpersonal relationships and surroundings. Moreso, participants felt that adjusting plans was an important aspect of managing their condition as it allowed them to take some degree of control over their IBD despite the unpredictable nature of their disease.

#### 3.1.4 Having a supportive medical team

Participants emphasized the positive impact of healthcare professionals who not only provided effective medical management but also offered emotional support. By developing a considerate and close-knit relationship with their medical team, participants felt assured that their providers had their best interest at heart, and this therefore allowed patients to feel a sense of confidence in utilizing the resources recommended to them for better outcomes.

#### 3.1.5 Feeling a sense of belonging

Participants emphasized the importance of feeling a sense of belonging, whether it be within their immediate social circles or through involvement in diverse support groups. This sense of connection in understanding how fellow individuals with IBD cope with their disease contributed immensely to the ability to navigate similar challenges.

A significant focus was also placed on the use of social media platforms, emphasizing the role these tools played in nurturing a sense of community and providing emotional support for addressing sensitive questions. Whether within online social groups or through active involvement in community forums for IBD, participants felt a sense of trust when actively contributing to conversations, particularly given their shared experiences.

### 3.2 Individual character strengths in IBD

#### 3.2.1 Staying determined despite setbacks

Participants demonstrated an unwavering mindset in the face of hindrances related to their IBD. They explained that developing a strong mindset to persist through physical challenges, such as abdominal pain and fatigue, was key to completing personal goals. Ultimately, participants viewed setbacks not as insurmountable barriers, but as challenges that could be overcome, highlighting their willpower to not let the disease restrict them from accomplishing their ambitions.

#### 3.2.2 Celebrating successes

Acknowledging accomplishments emerged as a notable attribute to promote resilience. Participants described the positive impact of recognizing their achievements, fostering a sense of triumph and motivation to continue overcoming setbacks that they otherwise thought were not possible with their condition.

Expressing gratitude was another significant aspect of resilience for individuals with IBD which underscores the importance of maintaining hopefulness while living with the disease. Ultimately, sustaining optimism allowed for the cultivation of a positive mindset encompassing thankfulness and appreciation for not only loved ones and caregivers, but for one’s life, even in the face of adversity.

#### 3.2.3 Having a sense of purpose

Participants who retained a clear vision of their medical objectives and personal aspirations exhibited greater resilience in navigating the challenges associated with IBD. Moreover, participants noted that deriving inspiration from their own actions or from others, such as having role models with IBD, provided them with a sense of tenacity to continue to overcome IBD-related challenges.

#### 3.2.4 Acceptance of the disease

Participants who came to terms with the changing demands of their condition demonstrated enhanced capability in managing both the emotional and practical aspects of treating their chronic illness. One participant shared her experience from reading a book by David Goggins, a renowned ultra-endurance athlete who manages a chronic illness himself, which served as a momentous inspiration to accept her disease. Goggins’ ability to persistently push his limits despite adversity related to his condition encouraged her to adopt a similar mindset toward managing her IBD and provided her with a strong sense of tenacity.

#### 3.2.5 Being kind to oneself

Participants emphasized the importance of treating themselves with patience, which also cultivated a more optimistic perspective on their journey with IBD. Recognizing that their condition might pose challenges at times, the ability to extend grace and kindness to themselves empowered participants to navigate obstacles with greater ease and understanding of their limitations.

#### 3.2.6 Belief in living a good life

Participants who maintained a determined outlook and compartmentalized their challenges were able to not only successfully overcome obstacles that seemed too great initially but were also able to maintain focus on life’s positive aspects. This enabled them to embrace the notion that leading a meaningful life was attainable and that their disease did not have to limit them from experiencing any normalcy in life.

#### 3.2.7 Capability of staying calm

Although many participants expressed feeling as though they were navigating through life in a state of “survival-mode,” the ability to remain calm during moments of anxiety proved pivotal in sustaining resilience. Furthermore, one participant observed a relationship between maintaining composure and a reduction in the physical discomfort associated with their disease.

### 3.3 Logistical strategies for resilience in IBD

#### 3.3.1 Proactive medical management

Participants emphasized the importance of adopting a proactive stance toward medical management and maintaining accountability in adhering to their medication regimen. They stressed the significance of attending regular check-ups, consistently adhering to medication schedules, and staying informed about available treatment options. These practical measures enabled individuals to take charge of their health and effectively navigate their medical journey.

#### 3.3.2 Moving on from challenges

Participants demonstrated a strong capacity to overcome challenges by leveraging their past experiences as valuable learning opportunities. Rather than dwelling on past hardships, participants embraced a forward-looking mindset, recognizing that the lessons garnered after adversity would ultimately serve them in the future. This adaptive outlook allowed them to navigate future challenges with greater confidence.

#### 3.3.3 Using stress-related strategies

In addition to mindfulness practices such as meditation and deep-breathing exercises, participants emphasized the importance of engaging in enjoyable hobbies. Whether it involved partaking in creative or communal endeavors such as reading, outdoor exploration, or socializing with friends, these activities served as effective coping mechanisms to alleviate stress. By proactively incorporating such methods into their lives, participants found that they were better equipped to maintain composure when confronted with overwhelming emotional or physical challenges related to their IBD.

#### 3.3.4 Capability to make changes in life

Participants demonstrated a readiness to adapt to their condition, whether it involved modifying their daily routines, dietary preferences, or social interactions. Some participants also highlighted the importance of incorporating regular exercise into their schedules and establishing structured agendas to remain accountable for these changes. Additionally, the willingness to switch medications, even on a frequent basis, was an important aspect that participants felt they had to embrace to manage their IBD.

#### 3.3.5 Recognizing when symptoms change

Participants emphasized the importance of being attuned to their body’s signals and quickly addressing any discomfort or unease. They elaborated on their practical approach to managing potential challenges by strategically preparing for emergencies. One participant mentioned assembling a portable kit containing necessary medications and tools to address unforeseen incidents related to their IBD. By proactively equipping themselves with essential supplies and being aware of their surrounding resources, participants could enhance their readiness to effectively manage unexpected situations.

## 4 Discussion

### 4.1 Interpretation of findings

Through a series of online focus group discussions, this qualitative study explored direct accounts from individuals with IBD on the unique elements that contribute to resilience in this patient population. Notably, participants emphasized prominence in cultivating resilience across three dimensions.

Interpersonal fortitude encompassed patients’ capacity to develop a supportive social and familial network to assist with the heavy emotional and physical demands of IBD. This was consistent with the literature findings from Bernhofer which highlighted the strong need for patients with IBD to utilize family as an important source of strength in helping patients manage their disease (Bernhofer et al., 2017). A previous study by Kumfer elucidated the notion that a patient’s environment could directly serve as either a buffer or exacerbate effects of stress (Kumpfer, 2002). Our study confirmed this conception in patients with IBD, as the participants in this study highlighted the effective use of establishing boundaries with others, including the option of distancing oneself from certain individuals or situations to reduce added stress resulting from those within their immediate environment. Additionally, utilizing social media to forge connections with fellow individuals coping with IBD contributed to reinforcing interpersonal resilience. As Goffman mentions, individuals who share similarities can create intimate social circles from which to obtain nonphysical support and feel a sense of acceptance (Goffman, 1963). Indeed, our findings revealed the proactive measures patients with IBD take to foster a sense of solidarity and assurance in their relationships by participating in activities with other individuals with IBD, either online or in-person. By partaking in such social events, participants felt empowered and comfortable seeking personal advice from others regarding their condition, a task they might otherwise have hesitated or felt embarrassed to undertake.

Individual character strengths that contributed to resilience among patients with IBD included establishing a strong sense of self and embracing one’s condition along with the challenges it presents. In a study conducted by Daniel et al., individuals with IBD often concealed their illness due to the perceived stigma associated with its symptoms (Daniel, 2002). However, participants in our study emphasized their capacity to overcome feelings of shame and fully embrace their condition. This acceptance empowered participants to “own” their disease, fostering a belief that they could lead a normal and healthy life despite the pain and magnitude of the challenges posed by their disease. Unique attributes of personal resilience in participants included the ability to celebrate milestones, whether personal or professional, which instilled a sense of pride within participants. Self-appreciation and the ability to trust themselves amidst the unpredictability of their condition facilitated greater self-awareness and emotional attunement. These are qualities essential for maintaining inner strength given the fluctuating nature of emotions often induced by the demands of patients’ unpredictable illness. Moreover, by extending grace to themselves during flares and maintaining composure in the face of adversity, participants experienced a sense of accomplishment in their ability to demonstrate patience while navigating their condition.

Participants demonstrated logistical resilience by taking ownership of their health, meticulously managing their disease through consistent medication adherence, and embracing necessary lifestyle and dietary modifications to enhance their well-being. Additionally, they employed tangible strategies such as cultivating a positive mindset and utilizing stress-reduction techniques to alleviate stressors. Our findings validate the resilience frameworks outlined by Keefer et al., encompassing behavioral interventions like mindfulness and cognitive diffusion techniques to enhance psychological adaptability, foster acceptance of the disease, and bolster self-efficacy (Keefer et al., 2021). Nutritional interventions, including diversifying dietary choices and minimizing food avoidance, were similarly instrumental in our study allowing participants to maintain resilience, consistent with Keefer’s resilience playbook (Keefer et al., 2021). Furthermore, adopting a positive mindset enabled participants to view physical challenges not as setbacks but as opportunities for growth, empowering them to recognize changes in symptoms and prepare accordingly.

Our study emphasizes that resilience, within the context of IBD, is not shaped by solely one component, but instead encompasses multiple factors to promote well-being. Positive-psychology based interventions involving the use of optimism and self-efficacy in addition to the use of resilience, to reinforce patients’ personal strengths can be critical for effective psychosocial care (Keefer, 2018). Indeed, participants in the study emphasized the critical role of these positive-psychology practices through their utilization of mindfulness techniques, such as daily meditation to prepare for the day, focusing on their breathing during moments of anxiety, and practicing gratitude even in difficult circumstances. Moreover, patients demonstrated optimism by perceiving adversity as a short-lived occurrence in life, further evidenced by their understanding and acceptance of the unpredictable demands of their condition. They expressed confidence in their ability to navigate challenges, viewing setbacks as opportunities for personal development. Self-efficacy (Keefer, 2018) was also evident in participants’ belief in their autonomous capacity to successfully manage tasks related to their disease. Such duties included the ability to make lifestyle changes such as adjusting medications or embracing new dietary restrictions, preparing for emergencies, and regulating emotions and attitudes toward their condition.

### 4.2 Practical applications

Utilizing a disease-specific resilience measure, as a patient-centered assessment, could potentially inform the customization of treatment approaches for individuals with IBD. For instance, if patients exhibit diminished logistical resilience yet strong individual character strengths, they might benefit from targeted advice on medically managing their condition, while recognizing their inherent emotional grit. Conversely, patients with limited individual character strengths and interpersonal resilience might find value in cultivating a positive outlook, practicing stress-management techniques, or honing skills to assert boundaries and improve communication with others. Through understanding patients’ strengths across each resilience domain, providers can decipher whether a treatment plan should focus more on psychological support, interpersonal building techniques, or medication and life-style adjustments.

A novel resilience-focused measure for IBD also has implications for policy development. To support the implementation of resilience-enhancement programs, policy makers can allocate financial resources to develop training workshops for healthcare providers to speak with their patients about the benefits of strengthening their resilience. Moreover, allocation can go toward building resilience-based support groups for patients in various settings. Many participants in our study conveyed their appreciation for the chance to share their journeys and connect with other individuals living with IBD through focus group discussions, which nurtured feelings of companionship and reassurance. Establishing comparable focus groups where patients can exchange their challenges and triumphs, and potentially offer guidance to those recently diagnosed, would foster a welcoming environment to deepen interpersonal connections among patients, thereby enhancing their interpersonal fortitude. Additionally, federal funding agencies have the capacity to offer novel grants to support research endeavors focused on exploring resilience within the IBD community. Such initiatives could encompass the development of intervention programs designed to enhance resilience with the overarching goal to advance quality of life and psychological well-being for patients living with IBD.

Lastly, integrating educational resources such as pamphlets, flyers, and online content for IBD patients is key for bringing awareness to this population on how to enhance resilience in their daily lives. Materials can include how to reduce stress during a flare, how to practice positive thinking when one is feeling doubtful or ashamed of their disease, and how to build a strong familial and social network, along with recommendations of webpages or public figures (e.g., role models) to follow for encouragement. In essence, patient education initiatives, comprising interactive workshops and webinars, are pivotal in aiding patients to cultivate positive-psychology techniques and problem-solving skills to enhance their resilience and bolster psychological health.

### 4.3 Limitations and strengths

This study had several limitations, one of which was its relatively small sample size. As most participants were in remission, this may have restricted the ability to capture critical insights from patients experiencing active symptoms, potentially affecting the comprehensiveness of the resilience factors identified. Another limitation was the use of purposive sampling that may have introduced selection bias, as participants were selected based on a predetermined criterion, therefore excluding individuals with lower resilience scores. Nonetheless, by intentionally selecting participants with moderate resilience scores, we ensured that patients could provide constructive definitions of resilience in IBD and comprehensively describe their coping mechanisms. Selecting patients with low resilience would have yielded potentially less rich information on both the definition of resilience and effective strategies, particularly if those patients used few or no coping strategies.

The research was performed in a specialized IBD clinic in New York City, with a sample predominantly consisting of Non-Hispanic, White, female patients with CD, likely limiting the study’s generalizability. Including more men and participants from various racial and ethnic backgrounds would enhance the scale’s applicability and relevance across different demographic groups. Moreover, while the study did include perspectives from patients with various IBD-related surgeries, it did not capture more complex disease manifestations (e.g., strictures, fistulas, perianal disease). Likewise, individuals without access to specialized facilities or those under the care of community gastroenterologists in less populated areas may use different coping mechanisms or have different experiences with their disease.

The heterogeneity of perceptions captured by the FGDs was essential in understanding resilience in IBD. Group interviews provided collective insights and promoted comparative reflection on resilience, while one-on-one interviews offered a more private setting for deeper individual exploration. As such, the qualitative approach was well-suited to uncover nuances of resilience that would be missed with solely quantitative methods. While recall and social desirability biases may have affected the credibility of proposed items, the candid FGDs confirmed existing resilience theories and revealed new dimensions. Moreover, the focus was on understanding how individuals identify resilience and navigate setbacks with IBD, even with imprecise recollections.

A final limitation was the cross-sectional design of the study, which captured resilience at a single point in time. A longitudinal study would provide a more comprehensive understanding of resilience dynamics, including patients who reported low resilience at enrollment but may have experienced higher resilience previously. Future research should aim to include patients with active and complicated disease manifestations, as well as post-surgical cases, to capture a broader range of resilience factors specific to these groups. This would ultimately strengthen the relevance of the scale for diverse patients and help mitigate selection bias, leading to a deeper exploration of resilience in IBD.

## 5 Conclusion

Through direct engagement with both patients and healthcare providers, this study represents a pivotal step toward advancing the understanding of resilience in IBD. The development of a drafted resilience measure for IBD lays an innovative groundwork that aims to support resilience-building efforts in this patient population. Moreover, the study highlights adaptive coping strategies, advocating for interventions rooted in positive psychology within IBD management. Future research will focus on the psychometric validation of the items chosen for the preliminary RISE-IBD. This will involve comparisons against existing resilience tools while considering other patient factors, particularly patient-reported outcomes, disease disability, and mental health scores.

## Data Availability

The original contributions presented in the study are included in the article/supplementary material, further inquiries can be directed to the corresponding author/s.
